# Individual-and community-level determinants of neonatal mortality in the emerging regions of Ethiopia: a multilevel mixed-effect analysis

**DOI:** 10.1186/s12884-020-03506-6

**Published:** 2021-01-06

**Authors:** Getayeneh Antehunegn, Misganaw Gebrie Worku

**Affiliations:** 1 Department of Epidemiology and Biostatistics, Institute of Public Health, College of Medicine and Health Sciences, University of Gondar, Gondar, Ethiopia; 2 Department of Human Anatomy, College of Medicine and Health Sciences, University of Gondar, Gondar, Ethiopia

**Keywords:** Ethiopia, Neonatal mortality, Emerging regions, Multilevel analysis

## Abstract

**Background:**

Unlike infant and child mortality, neonatal mortality has declined steadily in Ethiopia. Despite the large-scale investment made by Ethiopia to improve the health of newborns and infants, it is among the regions with the highest burden of neonatal mortality. Although there are studies done on neonatal mortality in different areas of Ethiopia, as to our search of pieces of literature there is no study in Emerging regions of the country. Therefore, this study aimed to investigate the individual and community-level determinants of neonatal mortality in the Emerging regions of Ethiopia.

**Methods:**

Using the 2016 Ethiopian Demographic and Health Survey (EDHS) data, secondary data analysis was done. A total weighted sample of 4238 live births in Emerging regions were included for the final analysis. A multilevel binary logistic regression was fitted to identify the significant determinants of neonatal mortality. The Intra-class Correlation Coefficient (ICC), Median Odds Ratio (MOR), Proportional Change in Variance (PCV) were used for assessing the clustering effect, and deviance for model comparison. Variables with a *p*-value < 0.2 in the bi-variable analysis were considered in the multivariable analysis. In the multivariable multilevel binary logistic regression analysis, Adjusted Odds Ratio (AOR) with 95% Confidence Interval (CI) were reported to declare statistically significant determinants of neonatal mortality.

**Results:**

The neonatal mortality rate in Emerging regions of Ethiopia was 34.9 per 1000 live births (95% CI: 29.8, 40.9). Being born to a mother who had no formal education (AOR = 1.79, 95% CI: 1.12, 2.88), being born to a mother who did not participate in making health care decisions (AOR = 1.25, 95% CI: 1.14, 1.79), and being twin birth (AOR = 6.85, 95% CI: 3.69, 12.70) were significantly associated with higher odds of neonatal mortality. On the other hand, being female (AOR = 0.67, 95% CI: 0.47, 0.95), having 1–3 Antenatal Care (ANC) visits (AOR = 0.34, 95% CI: 0.15, 0.74), high community media exposure (AOR = 0.64, 95% CI: 0.41, 0.98), and preceding birth interval of two to 4 years (AOR = 0.38, 95% CI: 0.24, 0.58) were significantly associated with lower odds of neonatal mortality.

**Conclusion:**

Neonatal mortality in Emerging regions of Ethiopia was unacceptably high. Maternal education, women’s autonomy in making decisions for health care, sex of a child, type of birth, preceding birth interval, ANC visit, and community media exposure were found significant determinants of neonatal mortality. Therefore, empowering women in making health care decisions and increasing access to mass media play a major role in reducing the incidence of neonatal mortality in Emerging regions of Ethiopia.

## Background

Globally, under-five mortality significantly decreased from 12.7 million in 1990 to 6.3 million in 2015 with 2.6 million died during the neonatal period [1]. It accounting for 40% of under-five mortality [2]. Approximately 98% of neonatal deaths occurred in low and middle-income countries [3, 4] and 39% in sub-Saharan African (SSA) countries [5]. The neonatal mortality rate has varied across countries ranged from 20 per 1000 live births in high-income countries to 31 per 1000 live births in SSA [6]. It is far below to achieve the Sustainable Development Goal (SDG) target of reducing the neonatal mortality rate of 12 or less per 1000 live births by 2030 [7, 8].

Despite the substantial decrease in child and infant mortality, the decline in neonatal mortality is steady [9]. As in most African countries, Ethiopia is one of the countries with the highest burden of neonatal mortality [10]. In Ethiopia, though child and under-5 mortality has dramatically decreased in the last two decades, neonatal mortality has steadily decreased [11]. According to the Ethiopian Demographic and Health Surveys (EDHSs) report, under-five mortality decreased from 166 per 1000 live births to 67 per 1000 live births, and infant mortality decreased from 97 per 1000 births to 48 per 1000 births, while neonatal mortality decreased from 49 per 1000 live births to 29 per 1000 live births, which is lower than under-five and infant mortality [12–14].

Infectious diseases, malnutrition, and birth complications are identified as the leading causes of neonatal mortality [2, 4, 15]. Previous studies conducted on neonatal mortality showed that residence [16], parity [17], educational status [17], mode of delivery [18], ANC utilization [19], birth interval [20], place of delivery [21], maternal nutritional status [22], and maternal obstetric factors [23] were statistically significant determinants of neonatal mortality. Neonatal mortality has significantly reduced in Ethiopia in the last two decades. However, it is marginally higher in Somalia, Afar, Gambella, and Benishangul-Gumuz regions [13, 24]. This highlights Ethiopia should work further to reach the Every Newborn Action Plan (ENAP) set a national target of less than 10 per 1000 live births by 2035 [25].

Though there are studies conducted on neonatal mortality in different areas of Ethiopia [26–29], there is limited evidence on the individual-and community-level determinants of neonatal mortality in Emerging regions. Therefore, this study aimed to investigate the individual and community level determinants of neonatal mortality in Emerging regions of Ethiopia using multilevel logistic regression analysis. Identifying significant individual and community-level determinants of neonatal mortality is crucial to reduce the incidence of neonatal mortality in Emerging regions of Ethiopia.

## Methods

### Study setting and design

The study used the 2016 Ethiopian Demographic and Health Survey (EDHS) data, which was obtained using a community-based cross-sectional study design. The 2016 EDHS is the fourth survey conducted in every five-year interval in Ethiopia. There are nine regional states (Afar, Amhara, Benishangul-Gumuz, Gambella, Harari, Oromia, Somali, Southern Nations Nationalities and People’s Region (SNNPR), and Tigray) and two city administrations (Addis Ababa and Dire Dawa) in Ethiopia. These regions are classified into three regions; emerging regions (Afar, Somali, Benishangul-Gumuz, and Gambella), developed regions (Amhara, Oromia, Harari, Southern Nations Nationalities and People’s Region (SNNPR) and Tigray) and two city administrations (Addis Ababa and Dire Dawa) [30]. A total of 13.1 million people of Ethiopia are living in Somali, Afar, Benishangul-Gumuz, and Gambella regions [31]. These regions are characterized by scattered pastoralists and semi-pastoral populations with extreme poverty and limited access to health care.

### Data source and sampling procedure

The data used for this study were retrieved from the most recent Ethiopian DHS survey (EDHS 2016). The EDHS is conducted every five-year using structured methodology and pretested validated standard tools to generate updated health and health-related indicators. The EDHS employs a multi-stage stratified cluster sampling technique to select the study subjects. In the first stage, a total of 645 Enumeration Areas (EAs) that represent the country were selected. In the second stage, systematic random sampling was employed and on average 28 households per EAs were selected. For this study, neonates born in Emerging regions of Ethiopia within 5 years preceding the survey were included. A total of 4238 neonates were used for analysis. The overall data collection and the sampling procedure was presented in the full EDHS 2016 report [13].

### Study variables

#### Outcome variable

The outcome variable for this study was neonatal death as reported by the mother, and it was defined as the death of a neonate within the first months of birth. This takes a binary outcome; such that neonatal death will be regarded as death (1 = if death occurs in the first month of life) or alive (0 = if the newborn alive in the first month of life).

#### Independent variables

The independent variable considered for this study were from two levels (at individual and community levels). At the individual, maternal age, marital status, religion, maternal education, paternal education, wealth index, maternal occupation, media exposure, maternal Body Mass Index (BMI)), number of ANC visit, the timing of first ANC visit, mode of delivery, preceding birth interval, place of delivery, women health care decision autonomy, size at birth, type of birth and birth order were included. At the community level, region, residence, community women education, community poverty, community media exposure, and distance to a health facility were considered. Community-level variables used in the analysis were from two sources; direct community-level variables that were used without any manipulation and aggregated community-level variables that were generated by aggregating individual-level variables at the cluster level.

Media exposure was measured from three variables such as reading the newspaper, listening to the radio, and watching television. These variables were merged and categorized as no “when there was no exposure to either of the three” and yes “when there was exposure to either of reading newspaper, listening radio and watching television”. Women’s health care decision making autonomy was assessed in EDHS, as a person decides on the respondent’s own health care. Which was categorized as women participating in making their own health care decisions and didn’t participate in making health care decisions (decides by their husband/partner). Birth weight was categorized as small, average, and large size at birth. Small size at birth is defined as birth weight less than 2500 g while birth weight greater than 4000 g is considered as large size at birth.

### Data collection procedure

The research was performed based on the 2016 EDHS data by accessing the data from the official database of the MEASURE DHS program www.measuredhs.com. For the study, we used the Birth Record (BR) data set.

### Data management and analysis

The variables were extracted from the BR dataset using STATA version 14 statistical software. The weighted data were used for analysis to adjust for unequal probability of selection and non-response. In EDHS, multistage stratified cluster sampling techniques were employed and the data were not flat. So, to draw valid inference and conclusion advanced statistical models such as hierarchical modelling, which consider independent variables measured at individual and community levels should be employed to consider the clustering effect/dependency. A two-level binary logistic regression model was employed to estimate the effect size of independent variables on neonatal mortality. Four models were fitted. The first model was the null model (a model without the explanatory variable), which was a model fitted to calculate the extent of cluster variability on neonatal mortality. It was assessed using the Likelihood Ratio test (LR), Intra-class Correlation Coefficient (ICC), Median Odds Ratio (MOR), and Proportional Change in Variance (PCV). The ICC is the proportion of total variance explained by the cluster variation [32].

ICC = σ^2^/(σ^2^ + π^2^/3)

Where *∂* ^2^ indicates that cluster variance.

MOR is the median values of the odds ratio of the cluster at high risk and cluster at lower risk of neonatal mortality when randomly picking two neonates from two clusters (EAs) [33].

$$ \mathrm{MOR}=\exp\ \left(\sqrt{\kern0.5em 2\ast \partial 2\ast 0.6745\kern0.5em }\right)\sim \mathrm{MOR}=\exp\ \left(0.95\ast \partial \right) $$

PCV is defined as the total variation of neonatal mortality explained by the final model (a model with individual-level factors and community-level variables) relative to the null model (a model without independent variables).

$$ \mathrm{PCV}=\underline{\operatorname{var}\ \left(\mathrm{null}\ \mathrm{model}\right)-\operatorname{var}\ \mathrm{full}\ \mathrm{model}}\left)\right) $$

Var (null model)

Model II (a multilevel model with individual-level variables); Model III (a multilevel model with community-level variables) and Model IV (a multilevel model adjusted with individual-and community-level variables) were fitted and a model comparison was made based on deviance.

Both bivariable and multivariable analyses were done. In the bivariable two-level binary logistic regression analysis, variables with a *p*-value ≤0.2 were considered in the multivariable analysis. The Adjusted Odds Ratio (AOR) with a 95% Confidence Interval (CI) in the multivariable multilevel analysis were reported to declare the statistical significance and strength of association between neonatal mortality and independent variables. By doing the pseudo linear regression analysis, the multi-collinearity was checked and the mean VIF was less than 5.

## Results

### Socio-demographic and economic characteristics of the mothers

A total weighted sample of 4238 neonates were used for this study. The majority (74.8%) of neonates were born to mothers with no formal education and 3997 (94.3%) of neonate’s mother were married. Of the total neonates, 3397 (80.2%) of their mothers did not have media exposure and 3082 (72.7%) of neonates were born to mothers aged 20–34 years. The majority (72.4%) of neonate’s mother was Muslim religion followers (Table 1).

**Table 1:** Socio-demographic and economic characteristics of mothers, 2016

Variables	Frequency (***N*** = 4238)	Percentage (%)
**Maternal age (years)**		
< 20	146	3.5
20–34	3082	72.7
35^+^	1010	23.8
**Maternal education status**		
No education	3170	74.8
Primary	764	18.0
Secondary and above	304	7.2
**Religion**		
Orthodox	352	8.3
Muslim	3068	72.4
Protestant	697	16.5
Others	121	2.9
**Wealth status**		
Rich	776	18.3
Medium	303	7.2
Poor	3159	74.5
**Marital status**		
Never married	9	0.2
Married	3997	94.3
Divorced/separated/widowed	232	5.5
**Women’s occupation status**		
Not working	2787	65.8
Working	1451	34.2
**Paternal education**		
No education	2476	58.4
Primary	800	18.9
Secondary and above	962	22.7
**Media exposure**		
No	3397	80.2
Yes	841	19.8
**Maternal BMI**		
< 18.5 kg/m^2^	1256	29.6
18.5–24.9 kg/m^2^	2450	57.8
≥ 25 kg/m^2^	532	12.6

*BMI* Body Mass Index, *kg* Kilograms, *m*^*2*^ Meter Square

### Child and maternal obstetric related characteristics

From a total of 4238 neonates, 52.5% were males and 68.2% were born at home. About 13.1% of the mothers had 1–3 ANC visits during their pregnancy and 2.8% were delivered through caesarean section. About 2.4% were twin births and 40.6% were large size at birth. Nearly three-fourths (70.2%) of the mothers were participated in making their own health care decisions (Table 2).

**Table 2:** Obstetric and health service related characteristics of respondents

Variable	frequency	Percentage (%)
**Place of delivery**		
Home	2894	68.2
Health facility	1347	31.8
**Mode of delivery**		
Vaginal	4118	97.2
Caesarean section	120	2.8
**Type of birth**		
Single	4134	97.6
Twin	104	2.4
**Birth order**		
First birth	769	18.2
2–4	1848	43.6
≥ 5	1621	38.2
**Preceding birth interval (in years)**		
< 2	1239	29.2
2–4	1748	41.3
> 4	1251	29.5
**Timing of first ANC visit**		
No ANC visit	3177	74.9
First trimester	354	8.3
Second trimester	672	15.9
Third trimester	35	0.9
**Number of ANC visit**		
No	3177	75.0
1–3	554	13.1
≥ 4	507	11.9
**Size of neonate at birth**		
Small	1031	24.3
Average	1488	35.1
Large	1719	40.6
**Women participating in making health care decisions**		
No	1264	29.8
Yes	2974	70.2
**Sex of child**		
Male	2223	52.5
Female	2015	47.5

*ANC* Antenatal Care

### Community-level characteristics of the mothers

About 85.1% of neonate’s mothers were from rural residents and 36.0% were in the Somali region. The majority (55.7%) of their mother was from a community with high poverty and 58.3% of the mothers reported perceived distance to visit health facilities as a big problem (Table 3).

**Table 3:** Community level characteristics of respondents, 2016

Variable	Frequency	Percentage (%)
**Region**		
Afar	1097	25.9
Benishangul-Gumuz	890	21.0
Gambella	724	17.1
Somali	1527	36.0
**Residence**		
Rural	3605	85.1
Urban	633	14.9
**Distance to health facility**		
Big problem	2472	58.3
Not a big problem	1766	41.7
**Community poverty**		
Low	1879	44.3
High	2359	55.7
**Community women** education		
Low	2429	57.3
High	1812	42.7
**Community media exposure**		
Low	2273	53.6
High	1965	46.4

### Neonatal mortality rate by respondent characteristics

**Fig. 1:**
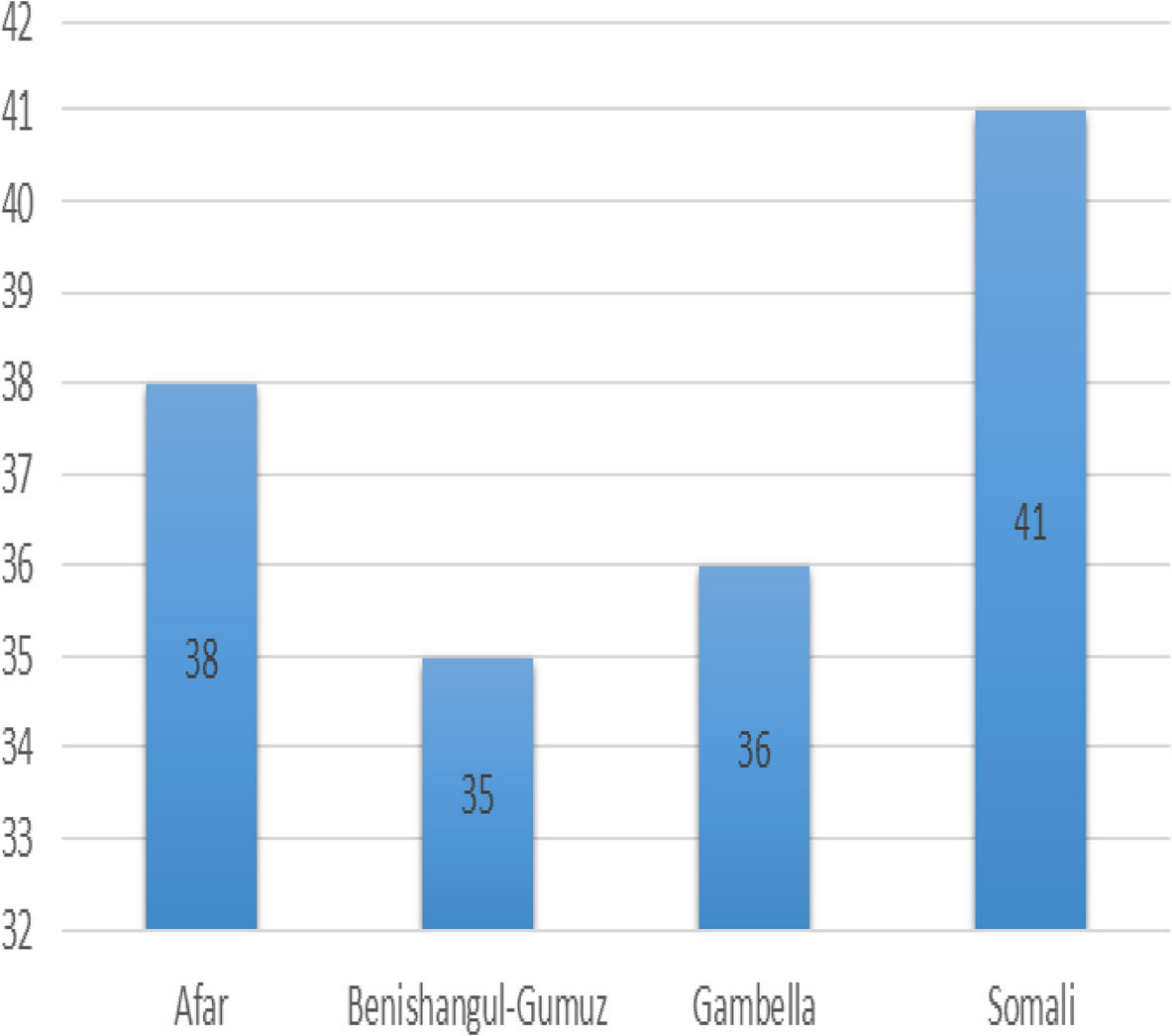
The neonatal mortality rates in Emerging regions of Ethiopia, 2016

The neonatal mortality rate in Emerging regions of Ethiopia was 34.9 (95% CI: 29.8, 40.9) per 1000 live births, which was highest in the Somali region (41 per 1000 live births) and lowest in the Benishangul region (35 per 1000 live births) (Fig. 1). The neonatal mortality rate among rural residents was 38.6 per 1000 live births (Table 4).

**Table 4:** Neonatal mortality rate by respondent characteristics, 2016

Variable	Neonatal mortality rate
**Residence**	
Urban	14.2
Rural	38.6
Wealth status	
Poor	39.6
Medium	26.4
Rich	19.3
**Media exposure**	
No	37.7
Yes	23.8
**Maternal age**	
< 20	47.9
20–34	30.8
35^+^	45.5
**Place of delivery**	
Home	38.4
Health facility	27.5
**Type of birth**	
Single	31.7
Twin	163.5
**Size at birth**	
Small	36.6
Average	31.6
Large	36.9
**Maternal education**	
No education	34.4
Primary	40.6
Secondary and above	26.3

### Determinants of neonatal mortality

#### Model comparison

The final model was the best-fitted model since it had the lowest deviance value. The ICC was 13.5% in the null model indicated that 13.5% of the total variability of neonatal mortality was due to differences between clusters/EA, with the remaining unexplained 86.5% was attributable to individual differences. Moreover, the MOR was 1.98 in the null model which indicates that there was variation between clusters, if we randomly select neonate from two different clusters, neonate at the cluster with a higher risk of neonatal mortality had 1.98 times higher odds of neonatal mortality as compared with neonate at cluster with a lower risk of neonatal mortality. PCV for the final model was 37.3%, indicated that 37.3% of the variability in neonatal mortality was explained by the full model (Table 5).

**Table 5:** Multivariable multilevel logistic regression analysis of neonatal mortality in emerging regions of Ethiopia, 2016

Variable	Null model	Model 1 (individual level factors)	Model 2 (Community level factors)	Model 4 (model with individual and community level factors)
**Sex of neonate**				
Male		1		1
Female		0.67 [0.47, 0.94]		0.67 [0.47, 0.94]^a^
**Wealth index**				
Rich		1		1
Middle		1.11 [0.44, 2.80]		0.87 [0.33, 2.27]
Poor		1.73 [0.91, 3.27]		1.22 [0.58, 2.55]
**Birth order**				
First birth		1		1
2–4		0.55 [0.28, 1.07]		0.62 [0.32, 1.21]
≥ 5		0.73 [0.36, 1.48]		0.82 [0.40, 1.66]
**Type of birth**				
Single		1		1
Twin		7.14 [3.84, 13.29]		6.85 [3.69, 12.70]^a^
**Preceding birth interval**				
< 2 year		1		1
2–4 year		0.36 [0.23, 0.56]		0.38 [0.24, 0.58]^a^
> 4 year		0.58 [0.32, 1.04]		0.64 [0.35, 1.17]
**Women participating in making their own heath care decisions**				
Yes		1		1
No		1.24 [1.12, 1.79]		1.25 [1.14, 1.79]^a^
**Media exposure**				
No		1		1
Yes		0.73 [0.42, 1.25]		0.97 [0.55, 1.73]
**Number of ANC visits**				
No visit		1		1
1–3		0.30 (0.14, 0.65)		0.34 (0.15, 0.74)^a^
≥ 4		0.44 (0.22, 0.88)		0.55 (0.26, 1.15)
**Place of delivery**				
Home		1		1
Health facility		0.77 [0.51, 1.16]		0.81 [0.54, 1.23]
**Maternal education**				
No education		1.66 [1.04, 2.65]		1.79 [1.12, 2.88]^a^
Primary		1.21 [0.53, 2.78]		1.50 [0.65, 3.46]
Secondary and higher		1		1
**Residence**				
Urban			1	1
Rural			2.04 [0.91, 4.54]	1.82 [0.75, 4.38]
**Distance to health facility**				
Not a big problem			1	1
Big problem			1.21 [0.83, 1.78]	1.16 [0.78, 1.72]
**Community poverty**				
Low			1	1
High			1.04 [0.67, 1.62]	0.85 [0.52, 1.39]
**Community media exposure**				
No			1	1
Yes			0.63 [0.41, 0.97]	0.64 [0.41, 0.98]^a^
Random effect				
Community level variance	0.51	0.41	0.37	0.32
Log likelihood	− 635.27	− 597.78	− 627.50	− 593.68
Deviance	1270.54	1195.56	1255.0	1187.36
ICC	13.5%	11.0%	10.0%	8.9%
MOR	1.98	1.84	1.78	1.72
PCV	ref	19.6%	27.5%	37.3%

^a^*ICC* Intra-class Correlation Coefficient, *MOR* Median Odds Ratio, *PCV* Proportional Change in Variance

In the multivariable multilevel analysis; maternal education, women who didn’t participate in making their own health care decisions, twin births, preceding birth interval, number of ANC visits, community media exposure, and sex of child were significantly associated with neonatal mortality. The odds of neonatal mortality among live births born to mothers who didn’t attend formal education had 1.79 (AOR = 1.79, 95% CI: 1.12, 2.88) times higher than live births born to mothers who attained secondary education and above. The odds of neonatal mortality among female births were decreased by 33% (AOR = 0.67, 95% CI: 0.47, 0.95) compared to male births. Being born to mothers who didn’t participate in making their own health care decisions were 1.25 (AOR = 1.25, 95% CI: 1.14, 1.79) times higher odds of neonatal mortality than births whose mother who participated in making health care decisions. The odds of neonatal mortality among twin births were 6.85 (AOR = 6.85, 95% CI: 3.69, 12.70) times higher compared to singletons. Furthermore, the odds of neonatal mortality among neonates in the community that had high media exposure were decreased by 36% (AOR = 0.64, 95% CI: 0.41, 0.98) compared to neonates in the community with low media exposure. The odds of neonatal death for neonates with preceding birth interval 2 to 4 years were decreased by 62% (AOR = 0.38, 95% CI: 0.24, 0.58) compared to neonates with preceding birth interval less than 2 years. The odds of neonatal mortality among children born to mothers who had 1–3 ANC visits during pregnancy were decreased by 66% (AOR = 0.34, 95% CI: 0.15, 0.74) than a child born to a mother who didn’t have ANC visit during pregnancy (Table 5).

## Discussion

Thousands of newborns die each year from preventable causes such as infectious diseases, malnutrition, and accidents, despite impressive success in reducing neonatal, infant, and child mortality in Ethiopia [34]. Neonatal mortality is the most sensitive indicator of limited health care access such as institutional delivery, vaccination, medical treatment of diseases, nutrition, and hygiene [35, 36].

This study found that the neonatal mortality rate in emerging regions of Ethiopia was 34.9 [95% CI: 29.8, 40.9] per 1000 live births. It was consistent with studies reported in the Jimma zone [26], and Nigeria [37]. However, it was higher than the 2016 EDHS report [13], and Afghanistan [38]. The possible explanation could be due to the present study was undertaken in Emerging regions (Somali, Afar, Gambella, and Benishangul-Gumuz) of Ethiopia where maternal and child health care services are relatively low and economically disadvantaged in contrast to other regions [39]. Furthermore, lower vaccine coverage reduced access to healthcare, poorly urbanized, and a comparatively high incidence of childhood infectious diseases such as malaria, and acute respiratory tract infections are found in Emerging regions compared to the other regions of the country [40, 41].

In the multilevel analysis; maternal education, preceding birth interval, type of birth, sex of neonate, community media exposure, number of ANC visits, and women participation in making health care decisions were significantly associated with neonatal mortality. A neonate born to mothers who do not have formal education had higher odds of neonatal mortality than a neonate born to mothers who attained secondary education and above. It is in line with studies reported in Sudan [42], Bangladesh [22], and Nigeria [16]. This may be because uneducated mothers may not have access to health information and less likely to visit maternal health care such as institutional delivery, ANC, and PNC [43, 44]. Another reason is uneducated mothers are reluctant to pursue childhood vaccination [45, 46] and more likely to practice prelacteal feeding [47], this could increase the risk of neonatal mortality. Besides, maternal education could result in good childhood feeding practices and have an improved awareness of common childhood disease preventive approaches that play a significant role in increasing newborn survival [48, 49].

In this study, being twin birth was a significant predictor of neonatal mortality. Twin births had higher odds of death in the first month of birth than singletons. It is consistent with the study finding in Ghana [17]. This could be since twin births are at higher risk of preterm delivery and fetal growth restriction and this could increase their risk of hypothermia, sepsis, and hypoglycemia that might increase the risk of neonatal mortality [50]. Neonates born within the preceding birth interval of 2 to 4 years had lower odds of dying within the neonatal period than those having a preceding birth interval less than 2 years. It was consistent with prior studies conducted in India [51], Afghanistan [38], and Indonesia [52]. The possible justification might be due to the reason that optimal birth spacing is vital for the health of the mother and newborn. The interbirth interval of 2 to 4 years could result in good pregnancy outcomes as women restore their nutritional and physiological loss from a previous birth, this could decrease their incidence of neonatal mortality.

Births to women who did not participate in making health care decisions had higher odds of neonatal death. This was in line with a study conducted in Bangladesh [53], it might be due to the reason that women who have participated in making health care decisions are more likely to use antenatal care service, gave birth at the health facility, and have a postnatal checkup in the early neonatal period, this could help to early identify danger signs of pregnancy and neonates and to seek medical treatment [54, 55].

The odds of neonatal mortality among female neonates were lower than male neonates. This was consistent with studies reported in Indonesia [52], and Nigeria [37]. This could be due to the sex differences in genetic and biological makeup, with males being biologically weaker and more susceptible to diseases and mortality [56]. Besides, the difference in mortality might be attributed to the different protein and gene expression variation in the placenta [57].

This study found that community media exposure was a significant predictor of neonatal mortality. Newborns from the community with high media exposure had decreased odds of death in the neonatal period than neonates from the community with low media exposure. This is in line with the study done in Bangladesh [58], the possible explanation might be the reason that mothers who have media exposure had better awareness of ANC utilization, institutional delivery, and childhood illness [59].

Newborns born to mothers who had 1–3 ANC visits during pregnancy had lower odds of neonatal mortality than newborns born to mothers who did not have ANC visits during pregnancy. It was consistent with studies in Kenya [60] and India [61]. This might be because a pregnant mother who had antenatal care visits receives health care such as iron, deworming, folic acid, and tetanus immunizations, this could decrease the risk of neonatal mortality. Besides, ANC creates an opportunity for mothers and newborns to receive different interventions such as anti-D, childhood vaccinations, and nutritional supplementation.

The strength of this study was the use of multilevel modelling taking into account the clustering effect in EDHS to draw valid inferences and conclusions. This study had limitations. As this stud was a cross-sectional study, it shares the limitations of cross-sectional study design. Besides, variables such as infectious diseases, sepsis, congenital anomalies, transplacental infections, HIV status, and medication use which are considered as the most common cause of neonatal mortality were not included in this study since it was not collected in EDHS 2016.

## Conclusion

Neonatal mortality in emerging regions of Ethiopia remains a major public health concern. Maternal education, women’s participation in health care decision making, sex of the child, type of birth, preceding birth interval, number of ANC visits, and community media exposure were significantly associated with neonatal mortality. Therefore, empowering women in education and their autonomy in making health care decisions as well as improving access to media plays a significant role in reducing neonatal mortality in emerging regions of Ethiopia. The government should scale up maternal and child health services in these regions to reduce neonatal mortality at the national level.

## Data Availability

Data is available online and you can access it from www.measuredhs.com.

## Abbreviations

ANC: Antenatal Care
AOR: Adjusted Odds Ratio, ARR
BMI: Body Mass Index
CI: Confidence Interval
COR: Crude Odds Ratio
CSA: Central Statistical Agency
DHS: Demographic Health Survey
EA: Enumeration Area
EDHS: Ethiopian Demographic Health Survey
ICC: Intra-cluster Correlation Coefficient
LLR: Log-likelihood Ratio
LR: Likelihood Ratio
MOR: Median Odds Ratio
PCV: Proportional Change in Variance
PHC: Population and Housing census
SNNPRs: Southern Nations and Nationality People Regional state
WHO: World Health Organization
