# Correction to: Individual-and community-level determinants of neonatal mortality in the emerging regions of Ethiopia: a multilevel mixed-effect analysis

**DOI:** 10.1186/s12884-021-03614-x

**Published:** 2021-02-05

**Authors:** Getayeneh Antehunegn Tesema, Misganaw Gebrie Worku

**Affiliations:** 1 Department of Epidemiology and Biostatistics, Institute of Public Health, College of Medicine and Health Sciences, University of Gondar, Gondar, Ethiopia; 2 Department of Human Anatomy, College of Medicine and Health Sciences, University of Gondar, Gondar, Ethiopia

**Correction to: BMC Pregnancy Childbirth (2021) 21:12**

**https://doi.org/10.1186/s12884-020-03506-6**

Following publication of the original article [1], the corresponding author would like to correct his name from Getayeneh Antehunegn to Getayeneh Antehunegn Tesema.

The original article has been corrected.
